# Grid Cells Encode Local Positional Information

**DOI:** 10.1016/j.cub.2017.06.034

**Published:** 2017-08-07

**Authors:** Revekka Ismakov, Omri Barak, Kate Jeffery, Dori Derdikman

**Affiliations:** 1 Rappaport Faculty of Medicine and Research Institute, Technion – Israel Institute of Technology, 1 Efron Street, Haifa 31096, Israel; 2 Network Biology Research Laboratories, Technion – Israel Institute of Technology, Haifa 32000, Israel; 3 Institute of Behavioural Neuroscience, University College London, 26 Bedford Way, London WC1H 0AP, UK

**Keywords:** grid cells, place cells, entorhinal cortex, hippocampus, cognitive map, spatial variability, remapping, spatial memory, path integration, self-localization

## Abstract

The brain has an extraordinary ability to create an internal spatial map of the external world [1]. This map-like representation of environmental surroundings is encoded through specific types of neurons, located within the hippocampus and entorhinal cortex, which exhibit spatially tuned firing patterns [2, 3]. In addition to encoding space, these neurons are believed to be related to contextual information and memory [4, 5, 6, 7]. One class of such cells is the grid cells, which are located within the entorhinal cortex, presubiculum, and parasubiculum [3, 8]. Grid cell firing forms a hexagonal array of firing fields, a pattern that is largely thought to reflect the operation of intrinsic self-motion-related computations [9, 10, 11, 12]. If this is the case, then fields should be relatively uniform in size, number of spikes, and peak firing rate. However, it has been suggested that this is not in fact the case [3, 13]. The possibility exists that local spatial information also influences grid cells, which—if true—would greatly change the way in which grid cells are thought to contribute to place coding. Accordingly, we asked how discriminable the individual fields of a given grid cell are by looking at the distribution of field firing rates and reproducibility of this distribution across trials. Grid fields were less uniform in intensity than expected, and the pattern of strong and weak fields was spatially stable and recurred across trials. The distribution remained unchanged even after arena rescaling, but not after remapping. This suggests that additional local information is being overlaid onto the global hexagonal pattern of grid cells.

## Highlights

• Individual grid cell fields exhibit large variability in firing rates
• The fields firing rate ratios tend to remain stable within and across sessions
• The fields' firing rate ratios are stable even during rescaling of the arena
• The firing profile of different fields is retained until remapping occurs

## Teaser

Ismakov et al. demonstrate that entorhinal cortex grid cell fields are more variable than previously thought and that this variability is stable between sessions, even if arenas are rescaled (but not during remapping). This suggests that grid cells store local positional information, in addition to their known hexagonal tessellation.

## Results and Discussion

Grid cells have multiple firing fields, organized in a hexagonal pattern spanning the entire environment [3]. These fields are equidistanced and similar in size and are generally considered to be uniform in amplitude [14]. Here we set out to test to what extent the fields are indeed uniform. We looked at several previous studies. First, a set of grid cells compiled from Bonnevie et al. [15], Derdikman et al. [16], and Sargolini et al. [17] were analyzed. These datasets contained entorhinal grid cell recordings of rats foraging in open field arenas, with the position of the animal and the firing activity recorded simultaneously (see STAR Methods). Since large variability in firing rates may arise from conjunctive grid × HD cells, which, in addition to exhibiting typical grid firing patterns, are also tuned to a given head direction [17], we eliminated such cells from the study. Since a portion of the cells in the dataset were recorded with only one LED positioned on the head of the animal, and thus head direction could not be determined, we used moving direction as an indicator of head direction. The moving-direction Rayleigh scores were strongly correlated with the head-direction Rayleigh scores for those cells in which we had the head-direction measure (r = 0.96; Figure S1A), providing us with a means to determine the head-direction conjunctivity of the cell with only one positioning LED present. Only cells with a low moving-direction Rayleigh score (<0.15) and a high enough gridness score (>0.3) recorded in noncircular arenas were included. In total, out of the 1,015 cells from the original datasets, 359 remained for analysis.

**Figure 1:**
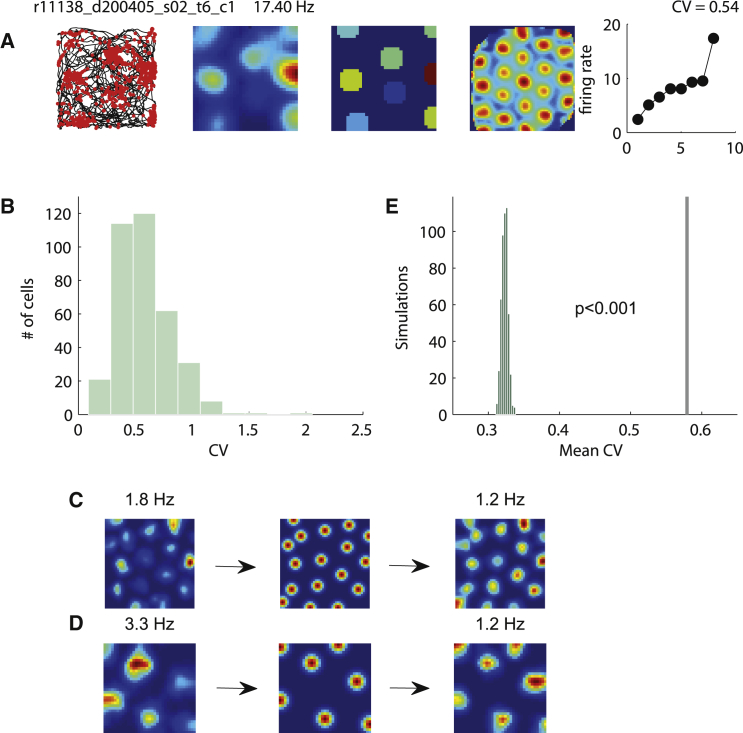
Grid Cells Exhibit Large Inter-field Firing Variability (A) Examples of (left to right) spike plot, rate map, zone map, autocorrelation map, and sorted field peak firing rate plot. Rate maps were generated by taking the number of spikes per second for each bin divided by dwell time in the bin. Zone maps were generated as a simplified version of the rate map, with only the fields' peak rate plotted, and were used for many of the analyses in the study. Sorted field peak firing rate plot displays the firing rates of all of the fields, plotted in increasing order. It was used to visualize the distribution of the firing variability. The coefficient of variation (CV) is indicated above. (B) Population results of all of the CVs, a measure of variability, used here to measure variability of field rates. (C and D) Examples of the rate maps of simulated spike trains. The left column is original rate map that was used to generate the rate map of equal rates (middle column). The right column is the rate map of a simulated spike train generated from the equal-rate rate map. The top example (C) was recorded in a 150 cm square arena, and the bottom example (D) in a 100 cm square arena. Values above rate maps denote maximum firing rates. (E) The distribution of mean CV from the simulated dataset. The real mean value is represented with a gray line (p < 0.002 from Monte Carlo, in comparison to simulated distribution). This large variability reveals that the firing rates among individual grid cells are not as homogeneous as generally assumed. See also Figures S1–S4.

We found a larger than expected variability between field firing rates derived from smoothed grid cell rate maps (Figure 1A; note that unless otherwise stated, we always used the value of the peak firing rate in the field). The majority of our grid cells possessed a coefficient of variation (CV; SD divided by mean) larger than 0.5 (58.2% of cells, N = 209/359; mean value of 0.579 ± 0.013; Figure 1B; we stress here that the CV is used here to measure variability of individual firing field rates).

In order to determine the probability of this occurring by chance, we repeated the analysis using simulated spike trains on the same rat trajectories. These spike trains were produced by means of contrived rate maps, which were used as probabilities of the cell discharging at a given location (see STAR Methods). The grid nodes used to produce these contrived rate maps were all equal in rate. The field locations remained in the same place as in the original cells, and the firing rate of individual fields was the same as that of the mean firing rate of the original cells (Figures 1C and 1D). The mean CV derived from the original data was larger than that derived from the simulated data for all 1,000 simulated runs (real mean = 0.579; median of simulated means = 0.319; p < 0.001, estimated from Monte Carlo simulated data distribution; Figure 1E).

We checked whether the running speed of the animal, which had previously been found to affect grid cell firing rates [17, 18], was the cause of the variability (if there were stereotypies in the way the rats explored the arena). We found no relation between the CV of the fields and the correlation of their rates to the mean speed of passage through them, suggesting that speed could not explain the variability (Figure S1B). We note that we found a negative relationship between the CV and the gridness of the cells, which is to be expected because a large difference between the fields can disrupt gridness to some extent (Figures S1C and S1H; r = −0.217; p < 0.0001).

We further checked whether overdispersion between individual passes through a given field (i.e., larger variability than expected by a simple Poisson spiking model) might account for the variability seen between the different fields [19]. To check this, we shuffled around the firing rate of each individual pass of the rat within the fields. If unreliability of firing was the cause of the variability as opposed to a change in the mean firing rate, the variability would remain high after shuffling. We found that this was not the case, with the CV being much higher in the real data compared to the shuffled data (p < 0.001 using the shuffling measure; Figure S4A). Therefore, overdispersion by itself was not able to account for the large variability between fields. In addition, temporal non-stationarity could not explain the spatial variability or stability in between sessions (Figures S4B and S4C). On the contrary—surprisingly, the stability of the field patterns seemed to be retained despite temporal non-stationarity.

**Figure 2:**
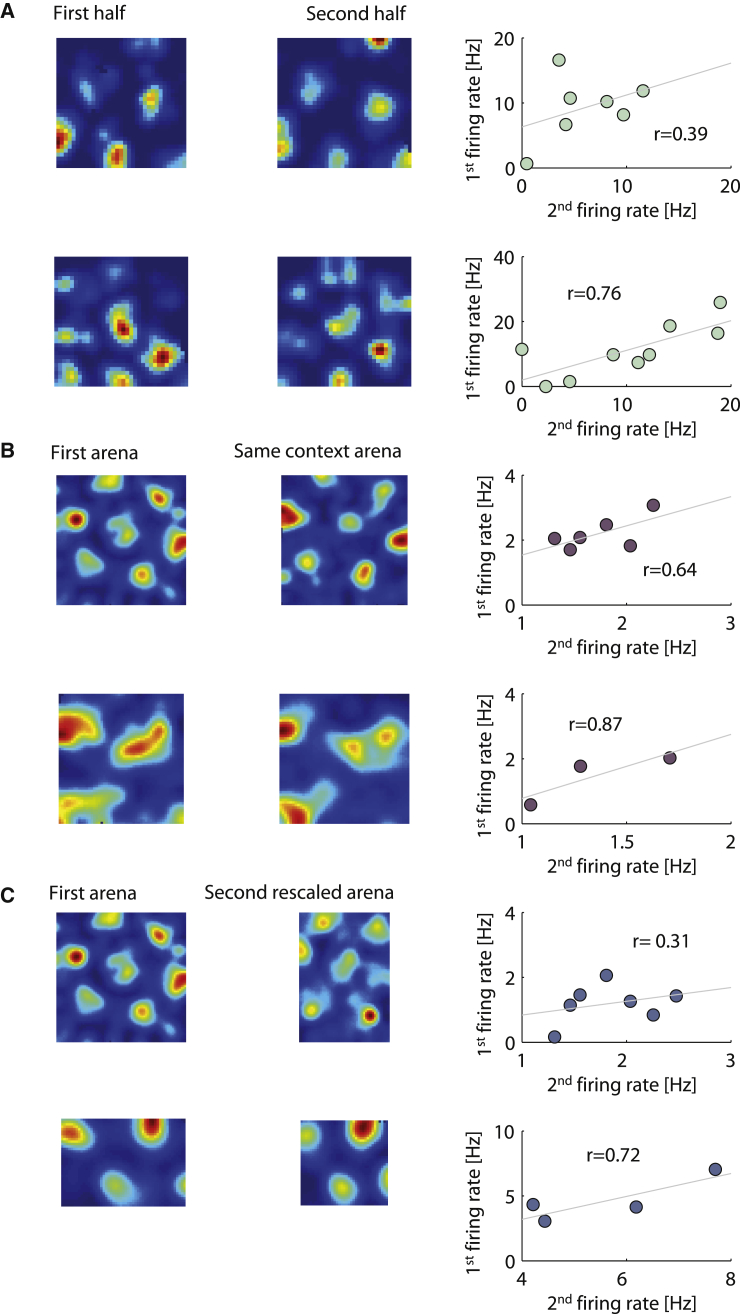
Examples of Firing Profile Remaining Stable Within and Across Sessions (A) Examples showing rate maps of sessions divided in half. A scatterplot of the peak firing rate between the fields is shown at the rightmost column, with the r value displayed in the plots. (B) Rate map and peak field firing rate scatterplots of first arena compared to same-context arena. (C) Rate map and peak field firing rate scatterplots of first arena compared to rescaled arena. This exposes that the differences in firing rates between grid fields remain stable across time.

**Figure 3:**
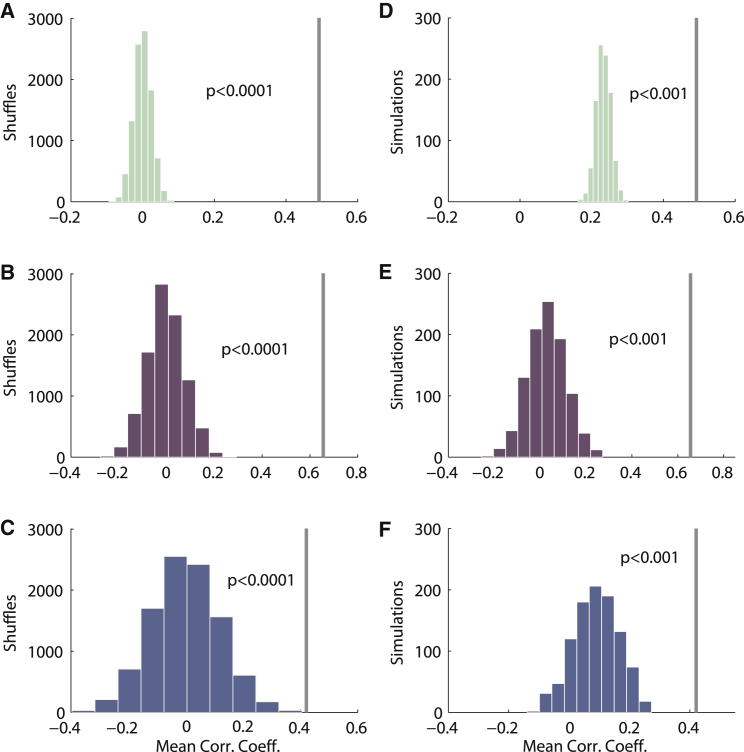
Population Results of Inter-field Firing Stability (A–C) Distributions of shuffled correlation coefficients between peak field firing rates of (A) split sessions compared to real value, (B) peak field firing rates of same-context sessions compared to real value, and (C) peak field firing rates of rescaling sessions compared to real value (p < 0.0001 for all, as derived from shuffling measures). (D–F) Distributions of simulated correlation coefficients between firing rates of (D) split sessions compared to real value, (E) peak field firing rates of same-context sessions compared to real value, and (F) peak field firing rates of rescaling sessions compared to real value (p < 0.001 for all, as derived from simulation measures). See also Figure S4.

We further wanted to assess how reproducible this firing variation was among the grid fields. We examined whether individual fields, specified by their spatial location, retained stable relative firing rates throughout single sessions and across arena changes. To observe this, we divided the single sessions in two and compared the firing rates of individual fields from the first half of the session with those of the corresponding fields in the second half. Corresponding fields were determined by overlaying the entire session field centers over the half sessions’ firing fields (see STAR Methods). We found examples of large correlation coefficients among the cells in the dataset (mean r value of 0.49 ± 0.02; Figure 2A shows typical examples; population results are discussed later in Figure 3).

To examine whether this result held true across sessions, we used data from Barry et al. [20] and Marozzi et al. [21]. In the Barry et al. dataset, grid cells were recorded in rescaled environments after the animal had been familiarized in a specific arena. The different arena configurations comprised a vertical south-north rectangular box, a horizontal west-east rectangular box, a larger square box, and a smaller square box, with most sessions usually starting with the larger square or vertical rectangle box. Each session consisted of four arena transformations, with the last arena matching the first [20]. The Marozzi dataset consisted of recordings of grid cells in five arenas of different contexts, alternating between different combinations of sensory cues, with either white or black colored walls and either a vanilla or lemon scent. Two of the five alternating arenas were recorded in the same context in order to confirm cell stability. For our analysis, we took the first and last sessions of the Barry et al. dataset and the same-context sessions of the Marozzi et al. dataset to estimate to what extent the inter-field firing rate stability persisted across different sessions in the same arena. Additionally, cells were only used if at least one of the arena pairs passed the criterion of a high gridness score (>0.3) and a low moving-direction Rayleigh score (<0.15), in order to ensure that it was a pure grid cell and was not tuned to head direction. In addition, we only took session pairs that had high 2D correlations between the locations of their firing fields (above 0.3, although we tried 0.2 and 0.4 without noticeable effects on stability), irrespective of firing rate of the fields: these were generated by simplifying the rate maps to include just the nodes of the grid pattern, which were then all equalized to 1, denoted from here on as zero-one zone maps (see STAR Methods and Figure S2). This was done to ensure that the cell’s grid pattern did not realign across same context sessions. Out of the total 144 cells (N = 69 from Barry et al. and N = 75 from Marozzi et al.), 47 cells passed these criteria and remained in the analysis (N = 17 and N = 30, respectively). Looking at the correlation coefficients of the firing rates of the first arena of the pair with the corresponding rates in the second same context arena, we also found examples of large r values (mean of 0.66 ± 0.05; Figure 2B; population results will be discussed later in Figure 3).

Additionally, we wanted to observe what happened to the inter-field firing stability when there was a change in the environment. Although the single sessions and the same-context sessions retained firing patterns in static environments, we were first interested in investigating how the stability would be affected when the arena rescaled in size. We used the Barry et al. dataset to check this. The original study found that in most cases, the grid tended to stretch or shrink with the resizing of the arena on average to about 80% of the size change. In addition to the high gridness score and low moving-direction Rayleigh score criterion, we additionally only accepted arena pairs in which the number of fields did not exceed a 30% difference between the two arenas. This was necessary to enable a clear matching of a corresponding field between the two environments, given the repeating grid pattern. Sixty-one arena pairs passed these criteria. We paired the fields by stretching the smaller dimensions to the size of the larger dimensions, in both the x and the y directions; the fields of the second arena pair that overlapped with the center nodes of the fields of the first arena pair were paired together. The correlation coefficients between rescaling arena pairs, as with the static environments, yielded examples of high values (mean r = 0.42 ± 0.07; Figure 2C). Overall, our analysis suggests there is a pattern that is retained between different environmental variations (Figures 2A–2C).

To determine the statistical significance of this finding, we compared the results to the distribution of a shuffling procedure in which the firing rates were shuffled between the different fields 10,000 times. Looking at the first half of the session versus the second half, the real data showed a larger mean correlation coefficient between the two halves than all 10,000 means generated from shuffled data (real mean = 0.49; median of shuffled data means = 0.00; p < 0.0001, estimated from shuffled population distribution; Figure 3A; also, mean slope = 0.513, which is higher than 500 shuffles, and mean intercept = 4.63, which is lower than 500 shuffles). The same-context arena pairs also yielded a larger mean correlation value compared to all 10,000 means generated from shuffled data (real mean = 0.66; median of shuffled data means = 0.00; p < 0.0001, estimated from shuffled population distribution; Figure 3B), as did the rescaling arena pairs (real mean = 0.42; median of shuffled data means = 0.00; p < 0.0001, estimated from the shuffled population distribution; Figure 3C).

We also compared the real results to the distribution of Monte Carlo simulation results, in which spike trains were simulated using the same rat trajectories, but assuming equal rates for all fields (see STAR Methods). Simulations of split session arenas yielded all lower means than the real value (real mean = 0.49; median of simulation-derived mean = 0.23, p < 0.001, estimated from Monte Carlo simulated distribution). The same was true for the same-context arena pairs (real mean = 0.66; median of simulation-derived mean = 0.03, p < 0.001 estimated from Monte Carlo simulated distribution), and rescaling arena pairs (real mean = 0.42; median of simulation-derived mean = 0.09, p < 0.001, estimated from Monte Carlo simulated distribution). This reveals that the inter-field variability is stable across sessions and that fields tend to retain a similar relative peak firing rate profile across time.

Previous studies found that the Fourier transformation of grid cells exhibits variability within the components that make up the grid firing pattern, which were concluded to lead to band-type firing within the firing pattern [22]. Using the grid components of the Fourier transformation, we reconstructed the rate maps, which were then used to calculate the CV. The CVs of the original data were much larger than those of the reconstructed data (Figure S3). Thus, the large CVs could not be explained by a non-uniformity of the Fourier components making up the grid [22] (Figure S3; see STAR Methods).

**Figure 4:**
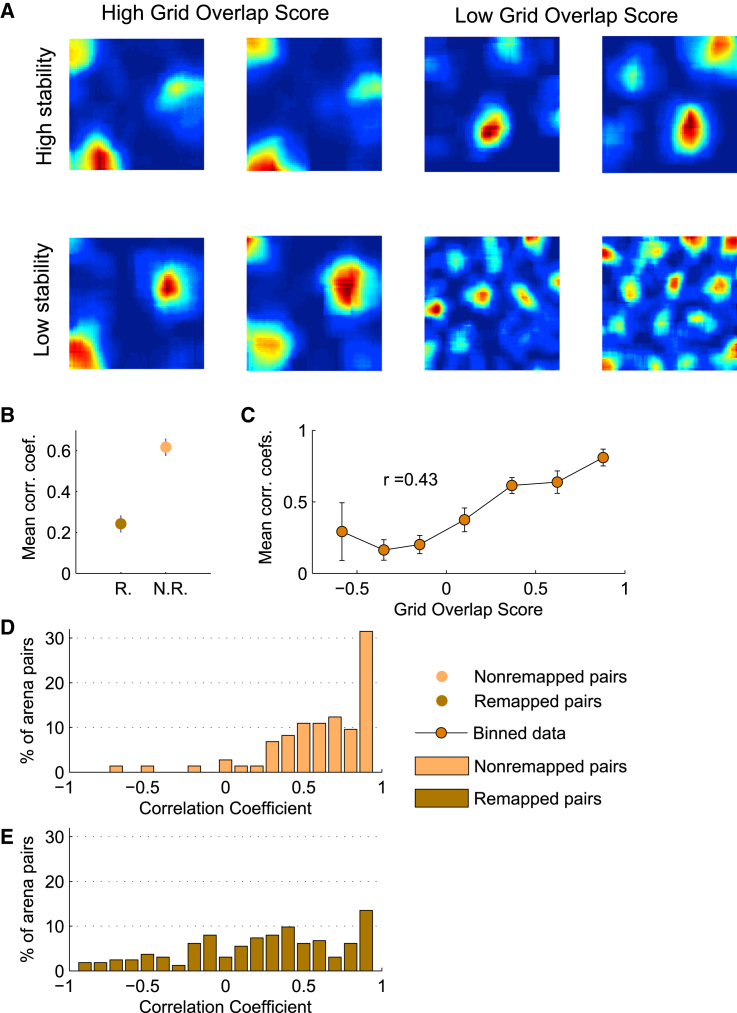
Firing Stability Is Lost with Grid Pattern Realignment (A) Rate map comparisons between split sessions, for cases with low and high grid overlap scores versus high and low firing stability. (B) Mean correlation coefficient between firing rates of corresponding fields in sessions exhibiting remapping compared to sessions exhibiting no remapping (non-remapping mean r = 0.63 ± 0.04, remapping mean r = 0.27 ± 0.04). (C) Plot of binned data showing mean correlation coefficient compared to the cross correlation gridness score of the zero-one zone maps (a measurement of grid realignment, irrespective of firing rates). The trend shows a positive correlation (r = 0.43, p < 0.001), indicating a larger correlation coefficient between firing rates with larger grid realignment. In short, the more stable the grid pattern, the more stable the firing rates among the fields across arena changes. Error bars indicate the SEM. (D) Histogram of correlation coefficients between firing rates across non-remapping arenas. Correlation coefficients are seen to be skewed toward 1. (E) Histogram of correlation coefficients between firing rates across remapping arenas. Correlation coefficients appear to be more uniform.

To look at whether this firing stability persists in different-context-induced grid remapping, we examined 234 arena pairs from the Marozzi et al. dataset, in which, as mentioned above, grid cells were recorded in different sensory context arenas by changing the color of the compartments (white or black), the scent of the arena (vanilla or lemon), or both. These changes are known to cause place cells to remap and thus might reflect a different encoding of local space. The changes resulted in grid translation, but not rotation. We separated the data into two categories, remapping (grid translated) versus non-remapping (grid not translated) patterns, and compared firing profiles. Remapping was determined by only looking at the location of the firing fields without taking peak firing rate into account by using the grid overlap score (Figure 4A). (Remapping pairs had an overlap score >0.3. We tried also 0.2 and 0.4 without noticeable differences.) Since grid translation would result in less overlap among the fields, we determined which fields corresponded to the previous arena fields by finding the minimum distances between their positions in both arenas, as opposed to overlapping the indices on top of each other, as done previously. We then looked at the correlation coefficients of the peak firing rates, finding that with no remapping, the rates remained static, but that the stability collapsed under grid remapping conditions (non-remapping mean r = 0.63 ± 0.04, remapping mean r = 0.27 ± 0.04; Figure 4B). Thus, conditions known to reorganize place cell encoding also altered the grid field firing rate spatial profile.

Additionally, the higher the grid overlap score, determined by the cross correlation gridness score of the zero-one zone-map pairs (see STAR Methods and Figure S2), the higher the mean correlation coefficient between the rates of corresponding fields tended to be (Figure 4C). Non-remapping arena pairs exhibited many more correlation coefficients around 1, revealing a higher propensity to retain firing stability when compared to the remapping condition (Wilcoxon rank-sum test, p value < 0.0001; Figures 4D and 4E). As a note of caution, we point out that it could have been that a different alignment of fields between the two remapped environments may have resulted in a better correspondence between field rates than we have found.

Our findings are thus threefold: (1) grid cells possess larger inter-field firing variability than expected by chance; (2) the grid shows a reproducible heterogeneity in rate that suggests local, as well as distributed, spatial modulation; and (3) grid cells react to context change by remapping their peak field firing rate distribution. These findings raise two questions: what causes the firing field heterogeneity, and what, if anything, might it be for?

As we have shown, heterogeneity between fields cannot be a result of overdispersion of the firing of individual fields or a result of speed modulation of the cells. Heterogeneity could stem from attractor dynamics within the grid population or from spatially modulated input to the entorhinal cortex. In the case of attractor dynamics, an activity manifold that has several peaks of different amplitude can lead to the observed effects, although it is not clear how such a difference in amplitudes between activity bumps could emerge in an attractor network without disrupting hexagonality. A corollary of this option is that the number of peaks in the manifold has to be large, as opposed to a single peak that translates periodically. Such a prediction can be tested by perturbing the grid network [23].

Inputs to grid cells can be of three major classes: feedforward from parahippocampal cortical regions, feedback from the hippocampal place cell system [24], or recurrent connections from other modules within the medial entorhinal cortex (MEC). Place information in the feedforward inputs has not been well described—some place specificity has been seen in retrosplenial cortex [25], but in general, input regions to the MEC are typically not known to carry place information [5, 26]. On the other hand, there are abundant feedback projections from place cells in the hippocampus to the deep layers of the MEC [24]. Inactivation of hippocampus causes grids to degrade [15], hippocampal replay during sleep leads grid cell replay [27], and place cells develop before grid cells [28, 29]. Previous models from our group and others have suggested that grids could be formed as a weighted combination of place cell inputs [30, 31, 32]. Given this, we suggest that it is plausible that the local non-uniformity in grid cell firing fields could be formed by increasing the projection strength to these grid cells from place cells in given regions of the environment, suggesting a possible interaction between projections from place cells and path integration in the formation of grid cells. Alternatively, projections from modules of larger scales could create a non-uniformity in the grid cells of a smaller-scale module. A third alternative is present in a recent study, finding similar results to ours, which suggests that the source of the variability is in sparse spatially selective input to the excitatory MEC cells, presumed to arise from external stimuli [33].

Another argument in support of the notion that grid field rate patterns derive from place cells is that after environmental manipulations, we saw grid cell responses that resemble those seen in place cells. Specifically, (1) similarly to place cells [34], deformation of the environment caused a shift of firing fields but no rearrangement of the place-specific component of the firing (i.e., the field locations, as reported by Barry et al. [20], and peak field firing rate profiles as seen in the present analysis); and (2) a change in the olfactory and/or visual context of the environments, known to cause global remapping in place cells [35, 36] and translation of grids [14, 21] caused, in our study, a reorganization of firing field rate profiles. These parallels are consistent with the notion that grid cells receive place-specific information from place cells (although they do not preclude the opposite—that place cells receive information from grid cells).

Does grid field peak firing rate heterogeneity serve a purpose? As mentioned above, it might simply be a byproduct of patchy excitatory feedback drive from place cells. Alternatively, perhaps it arises from these inputs but has a function, such as to contribute to the spatial separation of linked information like episodic memories [37]. However, an alternative possibility, given the abundance of MEC projections to the hippocampus [24], is that its function is to help drive place field spatial specificity. For example, a given place cell might respond to the drive from a given grid cell only when the rat is in the region of that cell’s strongest field, another when it is in either of the two strongest fields, and so on. With appropriate threshold setting, perhaps mediated by interneurons, the spatial firing rate pattern of a grid cell could thus help to uniquely specify a place.

A final possibility combines both the feedforward and feedback hypotheses. By this view, grid field firing rate heterogeneity derives from place field specificity, and the latter in turn derives from the former. Since grid field heterogeneity would necessarily arise from multiple place cell inputs, the projection back to place cells could provide a mechanism by which a given place cell could be informed about others simultaneously active in the network. In other words, grid cells could be a way for the system to accumulate the collected outputs of all the place cells active in that place and feed them back to individual place cells, thus allowing the system to combine focal place information and self-motion-related path integration signals, in order to derive an unambiguous estimation of current location.

## STAR★Methods

### Key Resources Table

**Table:** 

REAGENT or RESOURCE	SOURCE	IDENTIFIER
**Deposited Data**		

Sargolini dataset	[17]	http://www.ntnu.edu/kavli/research/grid-cell-data

**Experimental Models: Organisms/Strains**		

Long-Evans rats	[15, 16, 17]	Original data collected in these papers
Lister Hooded rats	[20, 21]	Original data collected in these papers

**Software and Algorithms**		

Custom MATLAB Code	This paper	https://github.com/derdikman/Ismakov-et-al.-Matlab-code
MATLAB 2014b	The MathWorks, Natick, MA, USA	https://uk.mathworks.com/products/new_products/release2014b.html

### Contact for Reagent and Resource Sharing

Further information and requests for resources should be directed to and will be fulfilled by the Lead Contact, Dori Derdikman (derdik@technion.ac.il).

### Experimental Model and Subject Details

All data used for this study were from previous published works, as follows:

The Bonnevie et al. dataset [15] were recordings from 8 Long-Evans rats, implanted chronically with two microdrives connected to four twisted platinum-iridium wire tetrodes. One of the microdrives was implanted into the MEC. For four of the rats, the second microdrive was implanted into the contralateral MEC, and for the other four, it was implanted into the hippocampus. The rats were trained to forage freely in a black square arena, 100 cm for six of the rats, and either 100 cm or 150 cm for the other two rats. See Methods section of [15] for more information.

The Derdikman et al. dataset [16] were recordings from 16 male Long-Evans rats, with implanted microdrives above the dorsocaudal MEC. Most rats also had an additional second microdrive in the dorsocaudal MEC above the other hemisphere. The rats were recorded in a free foraging task in 150 cm square arenas. See Methods section of [16] for more information.

The Sargolini et al. dataset [17] were recordings from 17 male Long-Evans rats, implanted with a microdrive connected to four platinum-iridium wire tetrodes above the dorsocaudal MEC. Additionally, five rats had a second microdrive implanted above the same location in the contralateral hemisphere. Rats were recorded freely foraging in familiar enclosures. For our analysis, we took the data recorded within the square arenas, which had walls of either 100 cm or 150 cm. See Methods section of [17] for more information.

The Barry et al. data [20] were recordings from six male Lister Hooded rats with electrodes implanted in the right dorsolateral MEC. Entorhinal activity was recorded as rats foraged for honey-sweetened rice in a four-sided enclosure placed in the center of the experimental room. After training in a given sized enclosure, the rats were recorded with the enclosure varying in size. Each recording session consisted of five rescaling arena sizes. See Methods section of [20] for more information.

The Marozzi et al. data [21] were recordings from eighteen male Lister Hooded rats with tetrodes implanted either in the MEC alone, or in both the MEC and hippocampal CA1. Fourteen of the rats were recorded in small context enclosures and seven in large (three were recorded in both). The enclosure was wiped repeatedly throughout the experiment with either lemon or vanilla food flavoring, with either white walls or black walls, allowing for four different context changes (white-lemon, black-lemon, white-vanilla, black-vanilla). Each recording session consisted of varying context changes. See Methods section of [21] for more information.

### Method Details

We only took the cells from the datasets that passed our gridness score and movement-directionality Rayleigh score thresholds (see below for more details, and Figure S1).

We used a diverse set of grid cells from multiple recordings and sessions compiled from multiple data sources [15, 16, 17, 20, 21]. Each grid cell received a gridness score that measures the strength of spatial periodicity, and a movement-directionality (MD) Rayleigh score that measures how tuned the cell is to head direction, as described below.

A firing rate map was produced by partitioning the arena into bins and taking the number of spikes that were fired per bin divided by time spent within the bin to calculate the dwell-time-normalized firing rate at each point within the environment. A bin size of 3-by-3cm was used. The rate map was then smoothed by a two-dimensional convolution of a Gaussian function of a standard deviation of sigma size 1.5 cm.

A gridness score was calculated by taking the correlations of rotational symmetry [17]. This was done by using the spatial autocorrelation maps of each rate map (as described in [17]), and then comparing it to centered rotated versions of itself at 30° intervals. The gridness score was then defined as the minimum of the higher correlation of the 60° and 120° rotation (*Acorr*_60°_, *Acorr*_120°_) from which was subtracted the maximum of the lower correlation of the 30°, 90°, and 150° rotations (*Acorr*_30°_, *Acorr*_90°_, *Acorr*_150°_):

$$Gridness\;score=\text{min}\left(Acorr_{60^{\text{°}}}\text{,}\;Acorr_{120^{\text{°}}}\right)-\text{max}\left(Acorr_{30^{\text{°}}}\text{,}\;Acorr_{90^{\text{°}}}\text{,}\;Acorr_{150^{\text{°}}}\right).$$

A HD Rayleigh score describes the strength of neuron’s head-directionality modulation. It is calculated by plotting the polar plot of the firing responses in relation to head direction. The length of the mean vector (Rayleigh vector) is then taken for the total circular distribution of firing rates. Since some of the cells only had one LED present that tracked head-direction, and thus the head-directionality could not be determined, we used movement-directionality instead, which is shown to be highly correlated with the head-direction based score (r = 0.96; Figure S1A).

### Quantification and Statistical Analysis

All correlation values, where given, are Pearson correlations. All ± values are standard-errors on the mean. All statistics are done using bootstrapping shuffling measures, or using simulations for the null hypothesis, as described in more detail below.

#### Inter-field variability analysis

Using the rate map and autocorrelation maps of the cells, the firing fields were located by finding the centers of firing from the rate map. The firing field radius was calculated as being 65 percent of half the distance between the center point of the rate map spatial autocorrelation map and the next closest field center peak. In addition, we calculated the number of firing fields, the peak firing rate of each field, and the grid orientation. The peak firing rate, as opposed to the mean, was chosen for the analysis in order to reduce potential artifacts arising from fields located near the borders, whose centers might be located beyond the boundaries of the arena. Also, the mean firing rate is dependent on how the place field size is defined, an issue that is resolved by using the peak rate.

In our analysis, we started by extracting these different parameters for each grid cell in our set. We created a “zone map,” by simplifying the rate map into place fields visualized by peak firing rate, with red representing high firing rate, and blue representing low rates (Figure 1A). We also plotted the firing rate of each field in increasing order, and used it to find the variability, and the CV between the firing fields of each cell.

“Zero-one zone maps” were created from the “zone map,” with all the field firing rates normalized to one, and the background equal to zero (Figure S2, part 1). This was to check grid realignment without taking the rate of firing into consideration. The point-to-point cross correlation score of the zero-one zone maps was used as a measure of grid realignment and termed the “Grid overlap score.”

#### Spike train simulation

Simulated spike trains were produced for each grid cell by using a computer-generated rate map with 2-D round Gaussian fields of equal size and equal firing rate. The maps were created by matrices of the same size as the rate-maps, with points at each of the centers (the amplitude of the point was multiplied by a constant such that the mean rate of the cell remained similar to the original after smoothing), and then smoothing these matrices using Gaussian smoothing with σ = 2 cm. Using different values for σ did not change the result substantially, as long as σ was in the order of magnitude of a typical grid field. We generated rate-modulated Poisson spike trains with the field centers at the same locations. The mean peak firing rate of the original rate map of the cell was used as the peak rates for the computer-generated rate map (Figures 1C and 1D). Spike trains were then simulated using the same trajectory of the rat laid over the generated rate map of similar magnitude fields. The full trajectory of the entire original session was used for the simulation. The analysis was then run again with the simulated spike trains, and simulated results compared to the actual data results.

#### Firing stability analysis

To check the stability of the firing profile across the full set of fields, we investigated three different conditions: first half of the session versus second half from the original open field data; first arena versus same context arena from the Barry et al. and Marozzi et al. data [20, 21]; and first arena and rescaled arena from the Barry et al. data [20].

Looking at the first half versus second half of the sessions, we found the centers of the firing fields from the entire session rate map, which we then used to pinpoint the fields in the first and second half and compare the correlations between the peak firing rates between the two cases.

Looking at the first arena versus the same context arena, we found the field centers in the first arena, and used those coordinates to pinpoint the corresponding field in the last arena. The correlation coefficient was calculated using the peak rates.

Looking at the first arena versus the rescaled arena, we stretched the arenas to the size of the maximum dimensions of the two arenas. Cases were only taken if the difference in the number of fields was not more than 70% to ensure that each field had a corresponding field in the rescaled arena. We then found the corresponding pairs by using the coordinates of the first arena overlaid on the second arena, after stretching to the same size. We again looked at the correlations between the peak firing rates among the fields.

Local maxima were used to detect field centers. To filter out false centers, fields that were too close to each other (defined as less than 70 percent of the distance of the center of the autocorrelation map to the closest local maximum) had the lower firing field center removed (Figure S2, part 2). Different distance thresholds were used, all producing similar results.

When investigating remapping versus non-remapping cases, instead of overlapping first session coordinates on top of the second arena, we instead found corresponding fields through minimum distances. Fields with minimum distance between them were paired together, until all the fields had a paired field (extra fields in the case of unequal number of fields between the two arenas were discarded). We used this method in this case, as remapping arenas would generally have less overlap between them as compared to the non-remapping conditions.

#### Shuffling procedure

In cases in which two sessions of a single grid cell were compared, the peak firing rates were randomly shuffled among the different fields for the two sessions. The different analyses were all then repeated with the shuffled data 10,000 times, and the distributions of the mean of the entire dataset compared to the real value to check for statistical significance.

#### Overdispersion analysis and shuffling

We found the firing rate of each individual pass through a field. To determine whether the large variability in field firing rates was due to larger overdispersion in fields at the individual pass level, we shuffled the momentary firing rates among all the different passes through the different fields. For each pass, the momentary firing rate was calculated as the number of spikes during the single traversal of the field, divided by the length of time of traversal. After shuffling, the momentary firing rates were assigned randomly to other passages, and the new number of spikes was calculated as the time of traversal of the current field times the shuffled momentary firing rate. We then calculated the coefficient of variance (CV) of the mean firing rate between all the fields after the shuffle. We then compared the mean CV of the original dataset to the shuffled dataset (Figure S4A).

We note that the shuffling did not eliminate potential influences from overdispersion, but rather other differences between fields, assuming a similar overdispersion distribution for all fields. Therefore using this procedure we kept the overdispersion, while negating the differences in a priori rates between fields. We did this by calculating the rate distribution for all fields combined together, and then we distributed these rates randomly between the fields, thus not negating the overdispersion after shuffling.

We also checked a version of this analysis while taking into account the non-stationarity of the data in time (Figures S4B and S4C). For this, we grouped individual field passes into consecutive blocks of 10 passes, and shuffled each block only in-between itself.

#### Correlation of CV and speed-rate correlation

Since firing rate has previously been shown to be modulated by speed [3, 18], we wanted to examine whether the high CV was due to this speed-rate correlation. This could occur if typically some fields were traversed at a higher speed than others. To do this we first found the speed score of each cell. The speed score was taken as a measure of the cell’s modulation to speed. Then we calculated the speed-rate correlation as the correlation between the cells speed score and its mean firing rate. We investigated whether there was a correlation between the CV of the cells’ firing fields and its speed-rate correlation (Figure S1B).

#### Fourier transform analysis

To check that the variability was not the result of variability in the grid components of the Fourier transformation, we reconstructed the rate map using just the grid nodes of the Fourier transform and checked how the variability differed from the actual values. We padded the rate maps with zeros to size [256x256] [22]. We then took the Fourier transformation of the padded rate map and extrapolated the six points of the grid component, taking the Fourier information only around those peaks (see Figure S3 for more information). We then reconstructed the rate map using these Fourier grid components. We compared the CV between the original rate map and the reconstructed rate map to examine whether the grid components solely were able to explain the variability.

### Data and Software Availability

The MATLAB code used in the analysis of this paper can be found at https://github.com/derdikman/Ismakov-et-al.-Matlab-code. The Marozzi et al. data are available on request to K.J.

## Author Contributions

Conceptualization, R.I., D.D., K.J., and O.B.; Methodology, R.I., D.D., and O.B.; Software, R.I.; Investigation, R.I. and D.D.; Resources, K.J. and D.D.; Writing – Original Draft, R.I., D.D., and K.J.; Writing – Review & Editing, R.I., D.D., K.J., and O.B.; Supervision, D.D.

## Supplemental Information

Document S1. Figures S1–S4

Document S2. Article plus Supplemental Information
