# Supplementary material for: Grid Cells Encode Local Positional Information
Source: Curr Biol. 2017 Aug 7;27(15):2337–2343.e3. doi: 10.1016/j.cub.2017.06.034 (PMC5558037; doi:10.1016/j.cub.2017.06.034)
Supplement: Document S1. Figures S1–S4 [file mmc1.pdf]

Current Biology, Volume *27*

Supplemental Information

Grid Cells Encode Local Positional Information

Revekka Ismakov, Omri Barak, Kate Jeffery, and Dori Derdikman

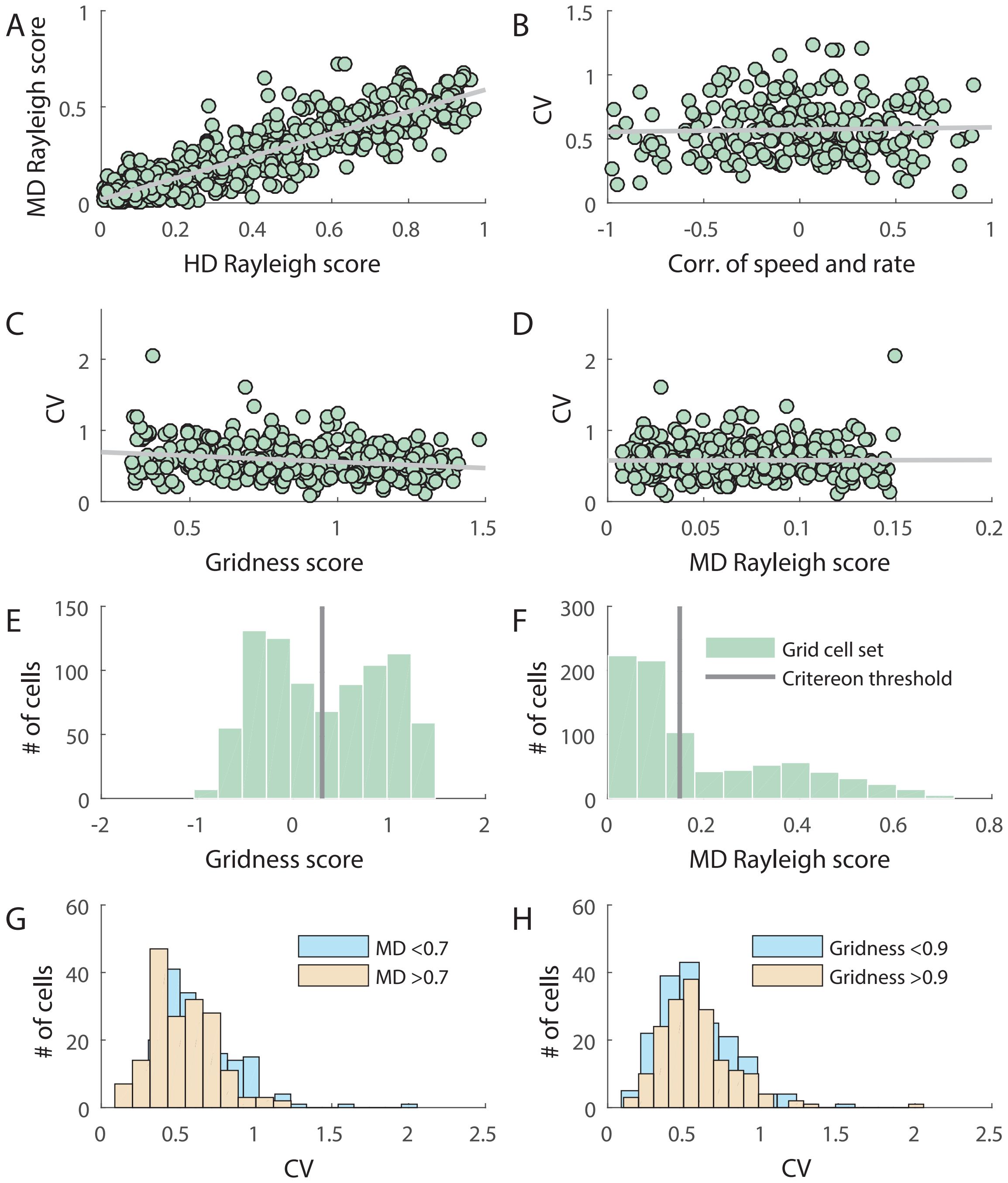

Figure S1. Cell statistics, related to Figure 1:

A) Scatterplot of head-directionality Rayleigh score versus movement-directionality Rayleigh score. High correlation shows that movement-directionality can be used as a strong indicator of head-directionality tuning in grid cells. This is beneficial since many cells in the data sets had only one LED present during recording, and thus head direction could not be determined.

B) Calculated CV as a function of the correlation between mean-speed and field rate, showing that CV is not determined by speed influencing the rates of fields. C) Calculated CV as a function of gridness scores for all cells in data set. D) Calculated CVs as a function of movement-direction (MD) Rayleigh scores for all cells. E) Histogram of all gridness scores of entire data set. Threshold of criterion cutoff indicated by gray vertical line. F) Histogram of all MD Rayleigh scores of entire data set. Threshold of criterion cutoff indicated by gray vertical line. G) Overlaying histogram of the CV of firing field peak rates of data split by MD Rayleigh score from data set used for analysis. Distribution similar between higher and lower MD scores. H) Overlaying histogram of the CV of firing field peak rates of data split by gridness score from data set used for analysis. Distribution similar between higher and lower gridness scores. This shows that threshold was set appropriately.

Part 1

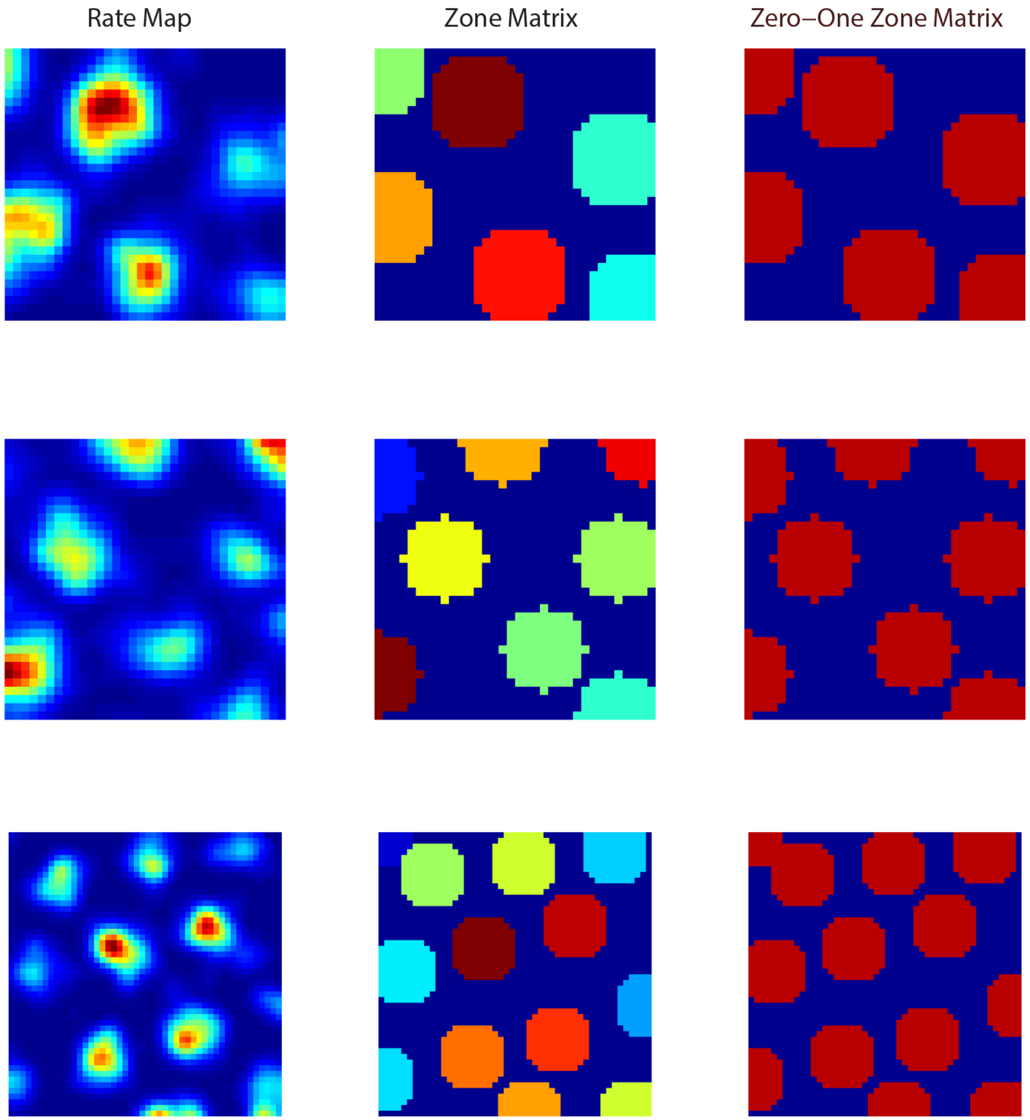

Part 2

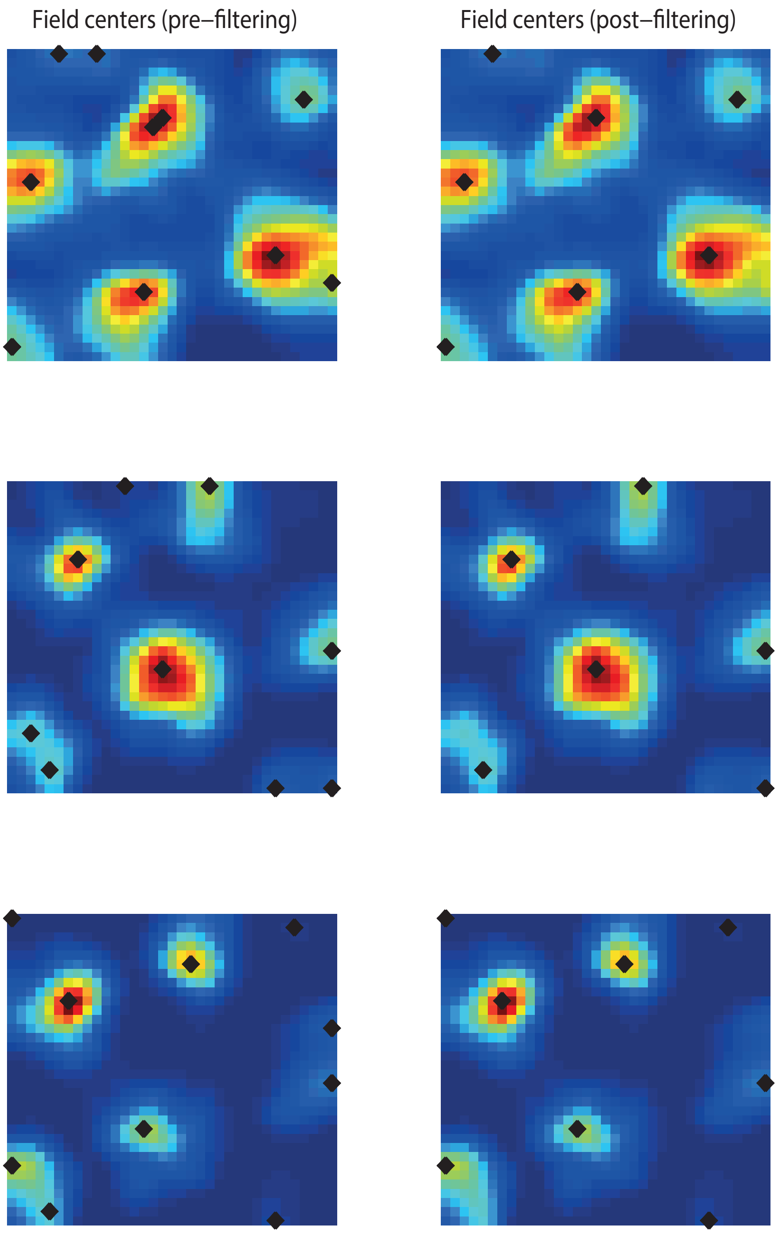

Figure S2. Analysis of single fields, related to figure 1:

Part 1: Rate maps and zone maps: Examples of rate maps (left column), zone maps (middle column), and zero-one zone maps (right column), for three cell examples. Part 2: Examples of finding the centers of fields: Fields were detected by finding the local maxima in the rate maps, with centers omitted if they were too close together (see Methods for more details). Left column shows the locations of the centers before omissions, and right column after omissions.

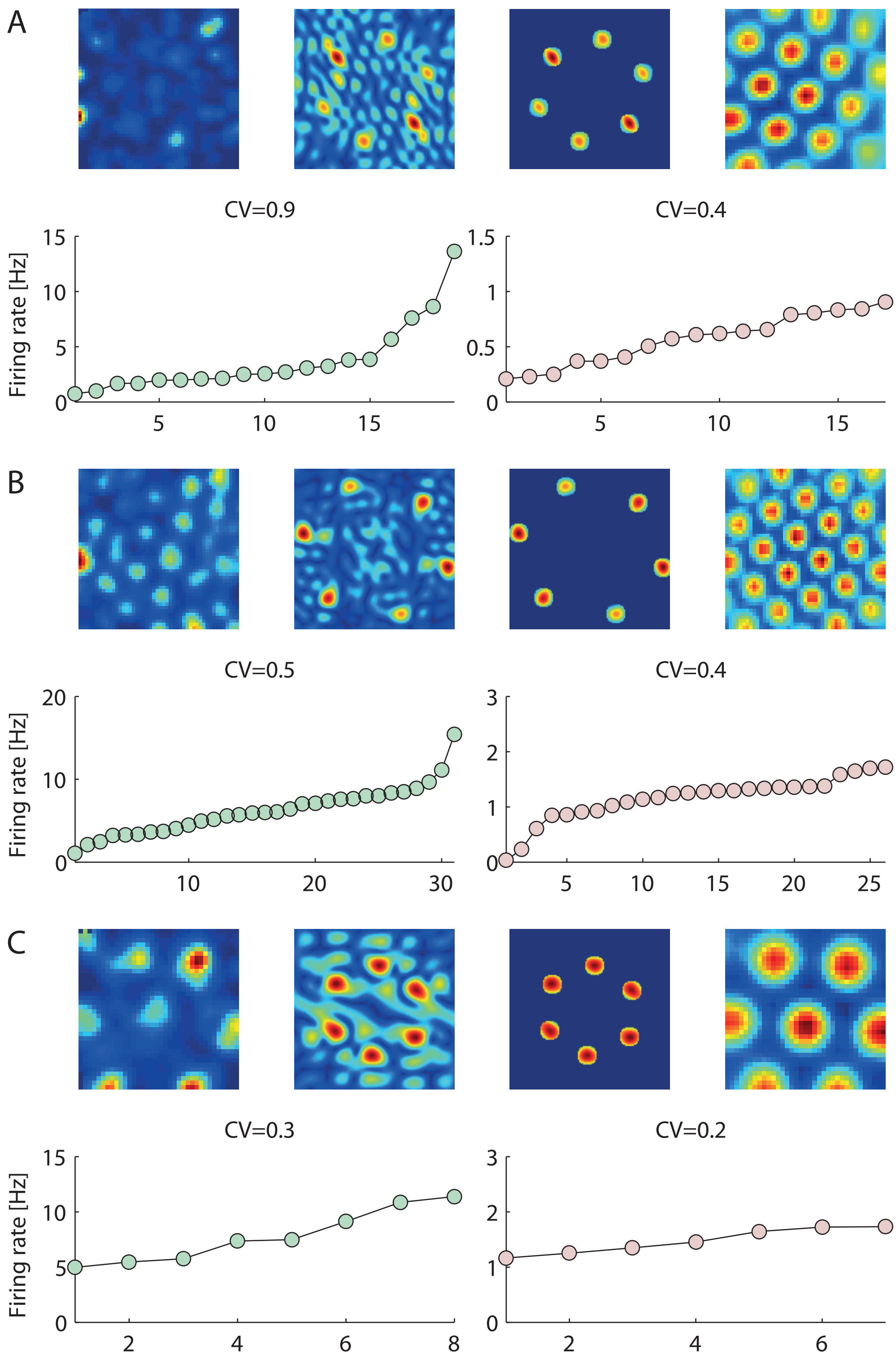

A
CV=0.9
CV=0.4
Firing rate [Hz]
B
CV=0.5
CV=0.4
Firing rate [Hz]
C
CV=0.3
CV=0.2
Firing rate [Hz]

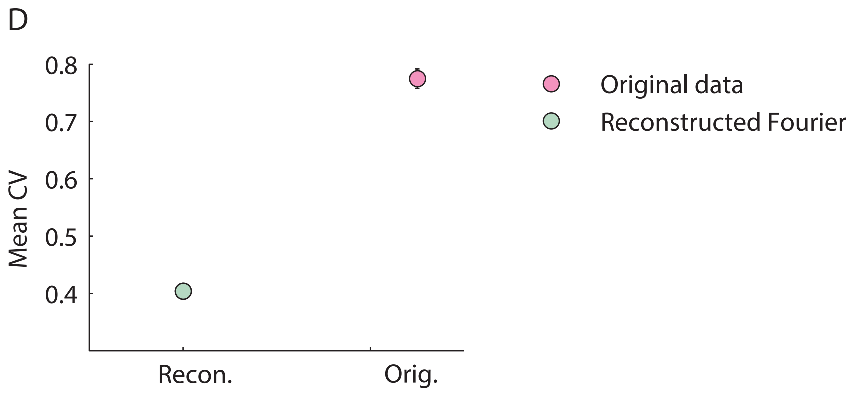

Figure S3. Variability is not due to Fourier non-uniformity, related to figure 1: A-C) Examples shown of rate maps (left-most image of each subplot) with the distribution of its fields' firing rates below, and the rate map reconstructed from the original rate map's Fourier transform grid components with its fields' firing rate distribution below. The Fourier transform and the extrapolated grid components of the Fourier transform are shown between the rate map and the reconstructed rate map. Distribution of firing field rates show greater variability within the original rate map than the reconstructed rate map's distribution. This shows that the variability of the cells cannot be attributed to the variability of the grid components of the Fourier transformation.

D) The mean CV of the cells compared to the mean CV of the reconstructed rate maps. The variability, measured by the CV, is larger for the original data than the reconstructed data. Fourier grid component variability cannot explain grid firing non-uniformity.

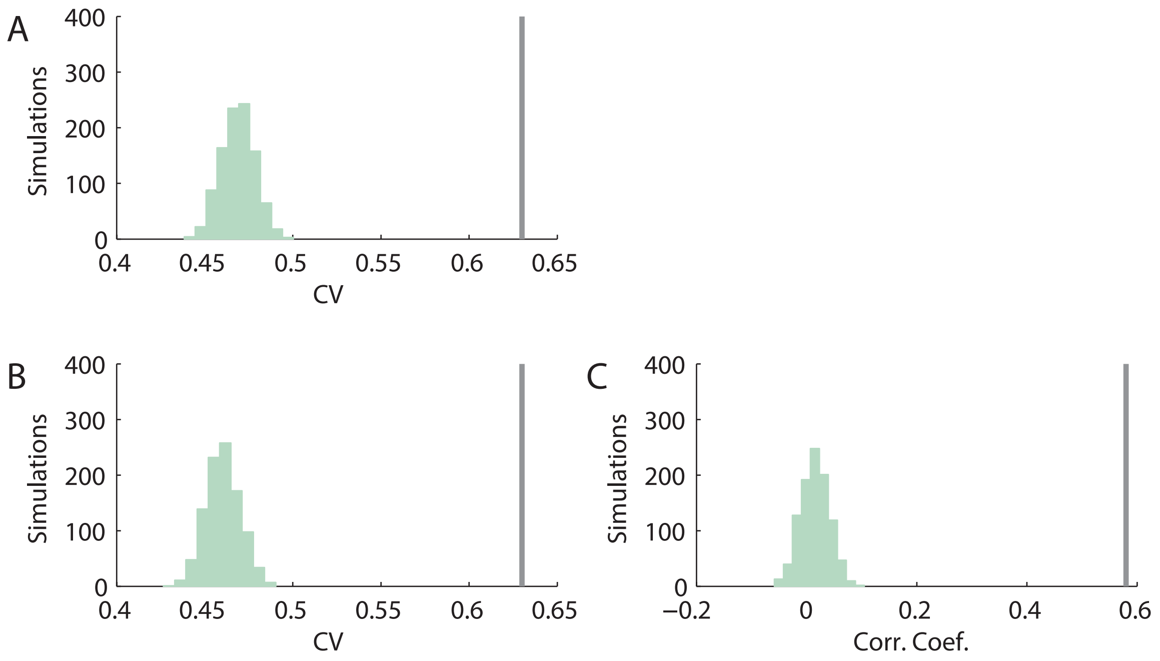

Figure S4. Overdispersion or temporal non-stationarity could not explain the results, related to figure 1 and figure 3:

A) The distribution of mean CV of the grid cell set after shuffling single-pass rates between individual passes through all fields. The real value of the mean CV without single-pass shuffling is indicated by the gray line ($p<0.001$, as derived from shuffling measure). Overdispersion between individual passes of the field is not able to explain the large CV. Note that while in figure 1 we looked at the peaks of each field, here the analysis is on the means of each field (because we are analyzing each single pass, we have to change the measure used).

B) The CV of the grid cell set after shuffling single passes only among consecutive blocks of 10 bins, as to take into account possible non-stationary effects of firing rate. Real value indicated by gray line ($p<0.001$, as derived from shuffling measure).

C) The correlation coefficient between the two halves of the sessions of the grid cell set after shuffling single passes in bins of 10 (similar to B), so as to only shuffle passes within the same temporal proximity. Real value indicated by gray line ($p<0.001$, as derived from shuffling measure).
